# Intrapancreatic bile duct metastasis from colon cancer after resection of liver metastasis with intrabiliary growth: a case report

**DOI:** 10.1186/s12957-015-0676-5

**Published:** 2015-08-21

**Authors:** Shoji Kawakatsu, Yuji Kaneoka, Atsuyuki Maeda, Yuichi Takayama, Yasuyuki Fukami, Shunsuke Onoe

**Affiliations:** Department of Surgery, Ogaki Municipal Hospital, 4-86 Minaminokawa-cho, Ogaki, Gifu 503-8502 Japan

**Keywords:** Intrapancreatic bile duct, Intrabiliary growth, Implantation, Metastasis

## Abstract

An extremely rare case of intrapancreatic bile duct metastasis from sigmoid colon adenocarcinoma is herein presented. Sigmoid colon cancer (T3, N0, M0, stage IIA) had been diagnosed and treated by sigmoidectomy in October 1993. In December 2002, a liver metastasis with intrabiliary growth was found, and this was treated by extended right hepatic lobectomy and caudate lobectomy with extrahepatic bile duct resection. In February 2014, intrapancreatic bile duct metastasis was found, and this was treated by subtotal stomach-preserving pancreatoduodenectomy. The intrapancreatic metastasis was judged to have arisen from cancer cell implantation, either by spontaneous shedding of cancer cells or as a complication of percutaneous transhepatic biliary drainage. Twelve months have passed since the last surgical intervention, and there has been no sign of local recurrence or distant metastasis. Differential diagnosis between intrahepatic cholangiocarcinoma and intrabiliary growth of a liver metastasis originating from colorectal adenocarcinoma is difficult but very important for determining the therapeutic strategy. Careful examination is needed to diagnose intrahepatic biliary dilatation, especially for patients with a history of carcinoma in the digestive tract and even if years have passed since curative resection of the digestive tract cancer. Aggressive surgical management for localized recurrence of a hepatic metastasis from colorectal adenocarcinoma may improve patient survival.

## Background

Intrabiliary growth of a liver metastasis originating from colorectal carcinoma is a rare manifestation of metastatic liver carcinoma [1] that resembles intrahepatic cholangiocarcinoma. Furthermore, intrapancreatic bile duct metastasis from a colorectal carcinoma is extremely rare, with few cases having been reported [2, 3]. Herein, we describe a case of intrabiliary growth of a liver metastasis that arose from a colonic carcinoma and was first diagnosed as an intrahepatic cholangiocarcinoma and then as a recurrent colon cancer when, years later, a recurrent tumor at the intrapancreatic bile duct was discovered, resected, and analyzed immunohistochemically.

## Case presentation

**Fig. 1:**
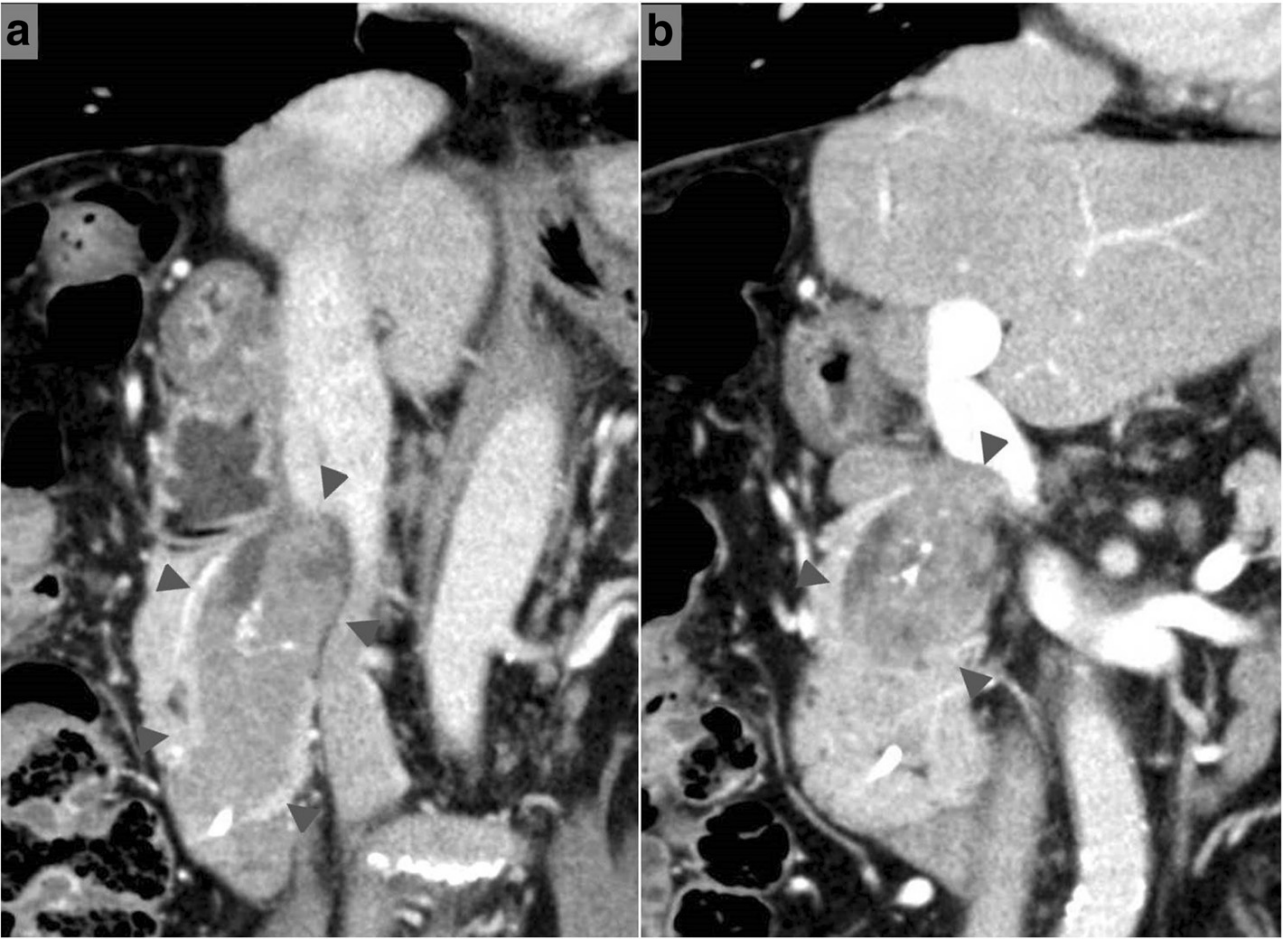
Computed tomography images obtained in February 2014. An obvious mass with clustered calcifications (*arrowheads*) in the dilated intrapancreatic bile duct (**a**) dorsal side (**b**) ventral side

A 73-year-old man was examined at our hospital in February 2014 for a chief complaint of epigastric abdominal pain. Computed tomography (CT) was performed and clearly depicted a mass with clustered calcifications in the intrapancreatic bile duct, which was dilated (Fig. 1). The patient’s medical history, which we describe below, led to a diagnosis of residual intrapancreatic bile duct cancer.

**Fig. 2:**
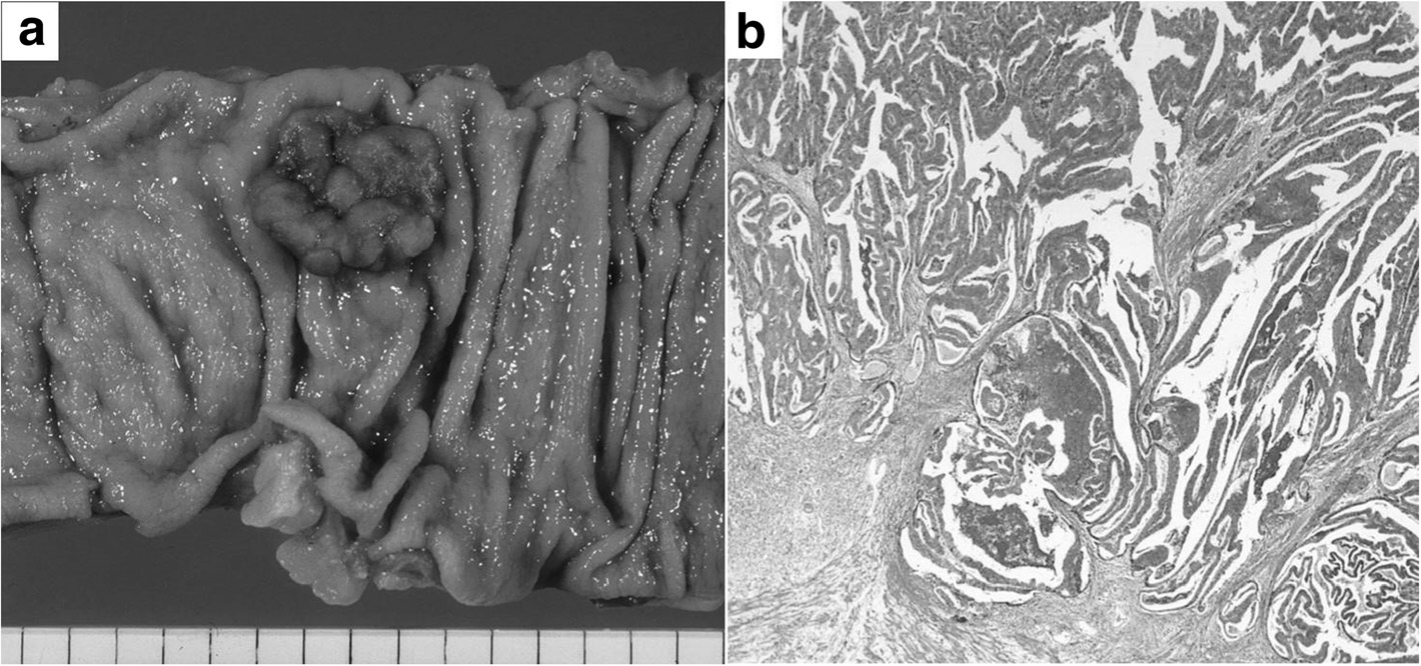
Macroscopic and histologic appearances of the sigmoid colon cancer resected in 1993. **a** A Borrmann type I tumor with a partial papillary structure was seen in the surgical specimen. **b** Histologic examination revealed a well- to moderately differentiated adenocarcinoma with papillary growth

**Fig. 3:**
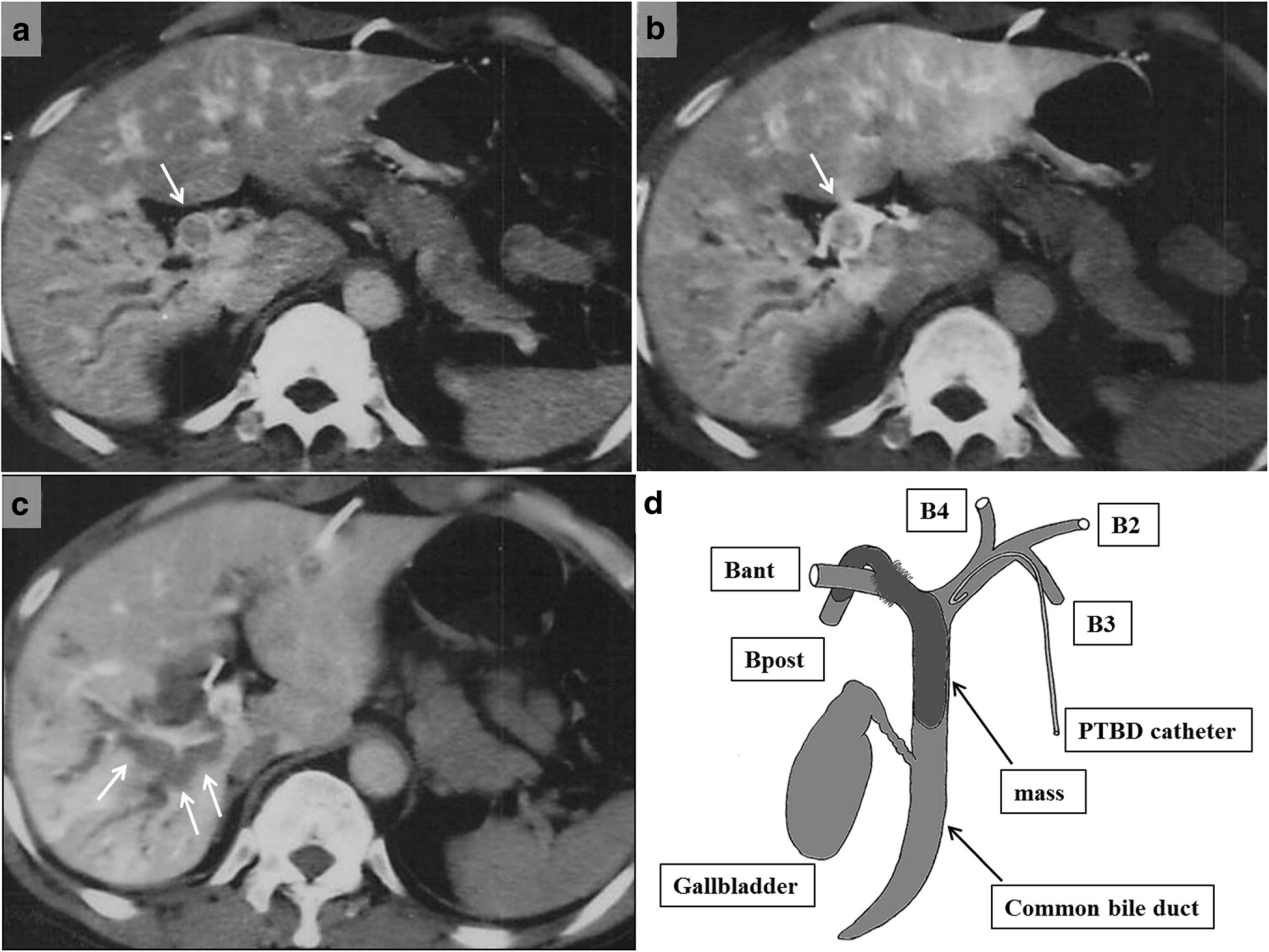
Follow-up contrast-enhanced computed tomography 9 years after sigmoidectomy. CT scans revealed **a**, **b** intrahepatic bile duct dilatation (*single arrow*) in the posterior segment of the right lobe to the common hepatic duct and **c** a low-density mass (*arrows*) in the dilated bile duct. A schematic diagram (**d**) shows the tumor location. *Bant* anterior right hepatic duct, *Bpost* posterior right hepatic duct, *B2* segment 2 bile duct, *B3* segment 3 bile duct, *B4* segment 4 bile duct

**Fig. 4:**
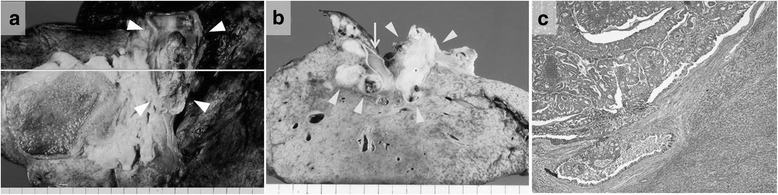
Macroscopic and histologic examination of the tumor resected in 2002. Macroscopic examination revealed **a**, **b** a papillary lesion (*arrowheads*) in the posterior segmental bile duct draining into the common hepatic duct. *Arrow* indicates the portal vein. Histologic examination revealed a well-differentiated adenocarcinoma (**c**) that formed an intrabiliary papillary tumor and invaded the liver at the posterior segmental bile duct to the right hepatic duct

The patient had undergone sigmoidectomy in October 1993 for a sigmoid colon cancer, which had the macroscopic appearance of a Borrmann type I tumor with a partial papillary structure and was 2.0 cm in diameter. Histologic examination of the surgical specimen revealed a well- to moderately differentiated adenocarcinoma forming the papillary structure (Fig. 2). The neoplasm was judged, according to the UICC TNM classification system, to be a stage IIA tumor that had infiltrated beyond the muscularis propria but without lymph node metastasis (pT3, N0, M0). No adjuvant therapy was performed, and the patient was followed up for 5 years with no evidence of recurrence. Nine years after the sigmoidectomy, obstructive jaundice developed, and contrast-enhanced CT revealed dilatation of the intrahepatic bile duct extending from the posterior segments of the right lobe to the common hepatic duct and a low-density mass within the duct (Fig. 3). Intrahepatic cholangiocarcinoma was diagnosed. Percutaneous transhepatic biliary drainage was performed, and this was followed by extended right hepatic lobectomy and caudate lobectomy with extrahepatic bile duct resection in December 2002. Roux-en-Y hepaticojejunostomy was performed, with the jejunal limb placed in the retrocolic retrogastric position. Macroscopically, the mass appeared as a papillary lesion in the posterior segmental bile duct that drains into the common hepatic duct. Histologic examination revealed a well-differentiated adenocarcinoma forming an intrabiliary papillary tumor and invading the liver at the posterior segmental bile duct that drains into the right hepatic duct (Fig. 4). Both the proximal and distal margins of the resected specimen were free of cancer cells. The postoperative diagnosis at that time was stage I intrahepatic cholangiocarcinoma (T1, N0, M0).

**Fig. 5:**
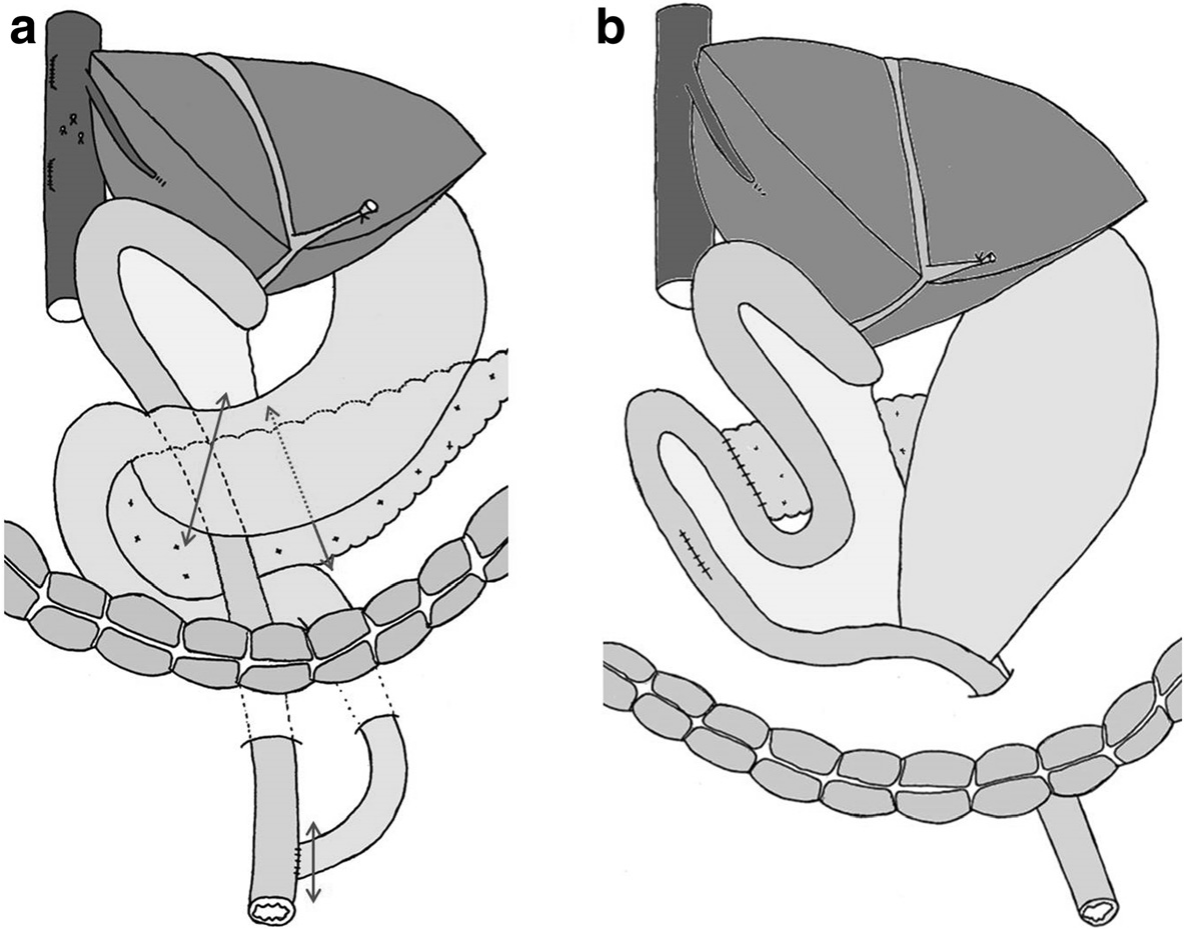
Schematic representation of the operative procedure performed in April 2014. **a** The dissection lines are marked by *two-headed arrows*. **b** Reconstruction after subtotal stomach-preserving pancreatoduodenectomy involved preservation of the previous bilio-enteric anastomosis

**Fig. 6:**
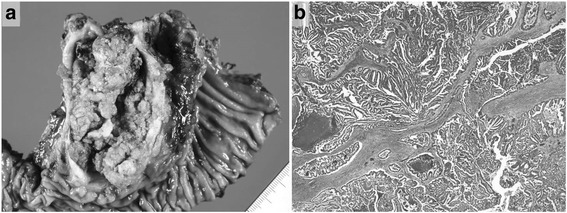
Macroscopic and histologic examination of the resected remnant intrapancreatic bile duct. **a** Macroscopically, a papillary lesion measuring 70 × 40 mm in size existed. **b** Histologic examination revealed a well-differentiated adenocarcinoma with slight invasion into the pancreas

**Fig. 7:**
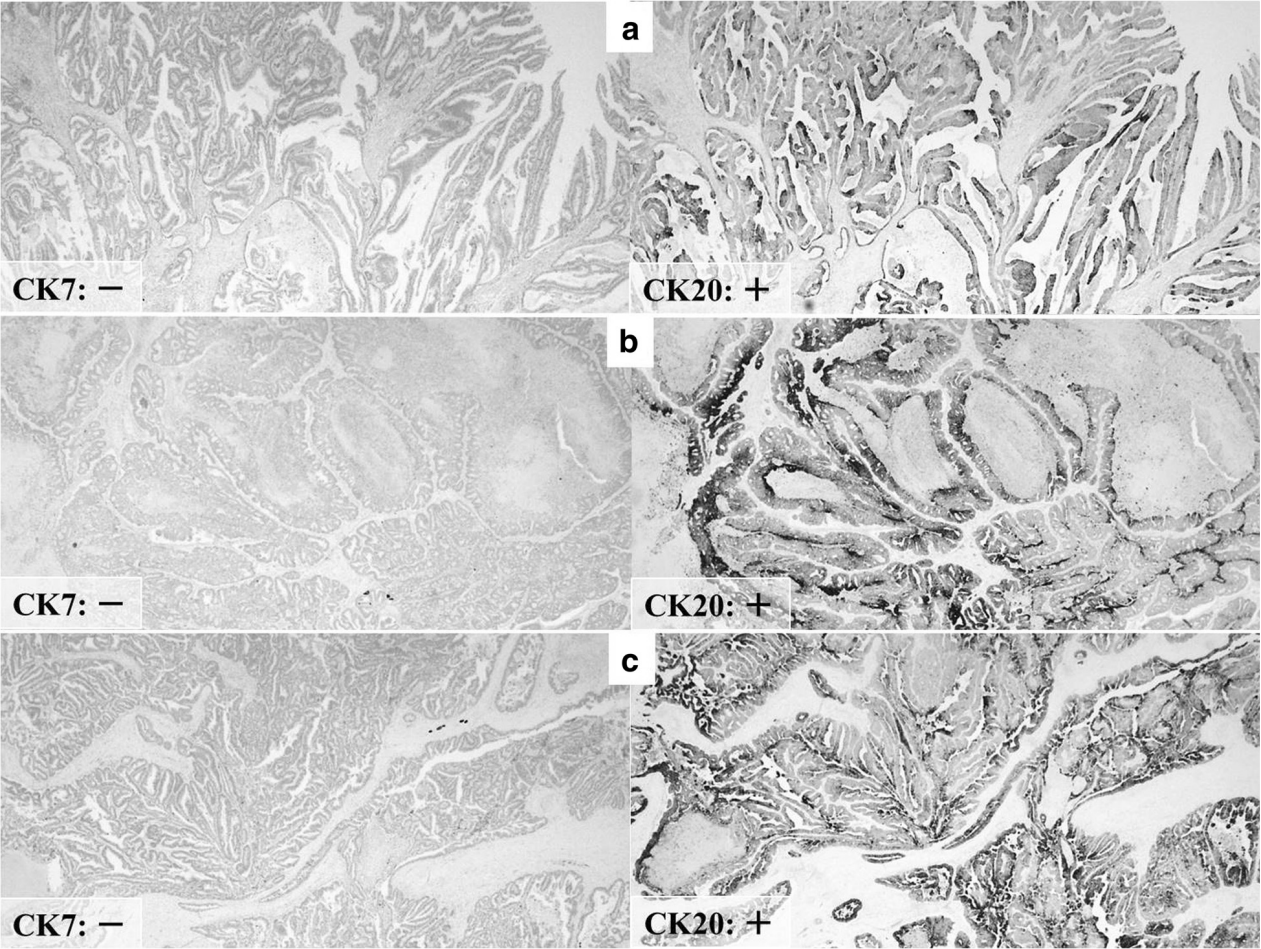
Immunohistochemical staining of all three surgical specimens showed similar staining properties. Sigmoid colon cancer resected in 1993 (**a**) was negative for CK7 and positive for CK20. The intrabiliary tumor in 2002 (**b**) and the intrapancreatic bile duct tumor in 2014 (**c**) were also negative for CK7 and positive for CK20

**Fig. 8:**
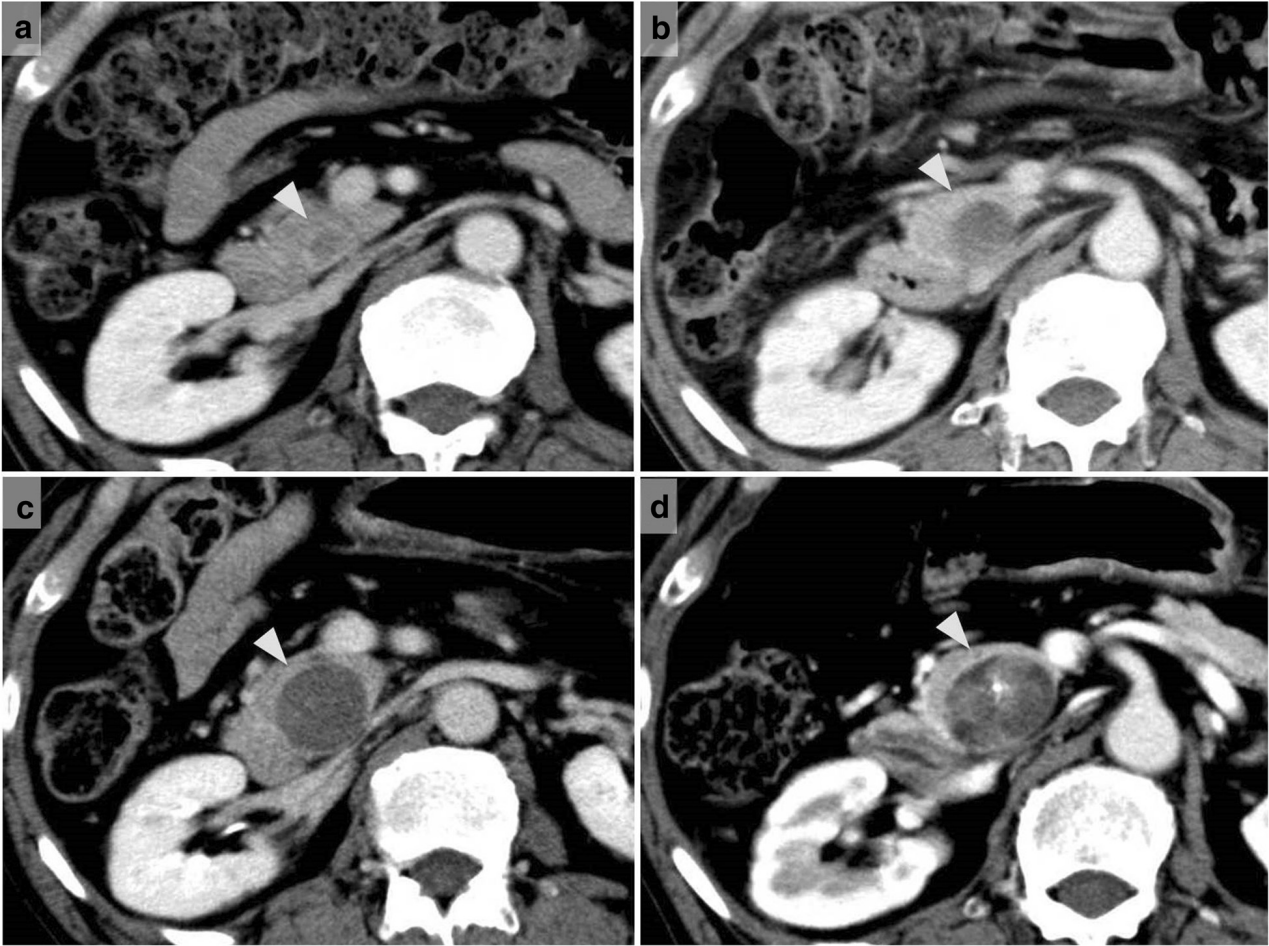
Contrast-enhanced computed tomography revealed the enlargement of the tumor in the intrapancreatic bile duct. CT scans obtained in **a** October 2005 (2 years and 10 months after hepatectomy) depicted a very low-density mass in the intrapancreatic bile duct. CT scans obtained in **b** December 2007 (5 years after hepatectomy), **c** January 2011 (8 years and 1 month after hepatectomy), and **d** February 2014 (11 years and 2 months after hepatectomy) show gradual dilatation of the intrapancreatic bile duct; the mass had gradually enlarged

The patient was followed up regularly after the hepatectomy, and at 2 years and 10 months, contrast-enhanced CT revealed intrapancreatic bile duct dilatation, but no cancerous lesion was detected, so the routine follow-up examinations were continued until February 2014, when the residual intrapancreatic bile duct cancer was discovered. The discovery was followed by performance of subtotal stomach-preserving pancreatoduodenectomy in April 2014. The previous bilio-enteric anastomosis was preserved (Fig. 5). The operation time was 229 min, and the blood loss volume was 630 mL. There was no intraoperative complication. However, a grade B postoperative pancreatic fistula (International Study Group on Pancreatic Fistula (ISGPF) classification) developed [4], and thus, the patient’s postoperative hospital stay was 30 days. Macroscopically, a papillary lesion measuring 70 × 40 mm was noted in the remnant intrapancreatic bile duct. Histologic examination revealed a well-differentiated adenocarcinoma with invasion into the pancreas but no lymph node metastasis. There was no evidence of lymphatic, vascular, or perineural invasion (Fig. 6). At this stage, the tumor was diagnosed as a remnant intrapancreatic bile duct cancer, but the histologic type, based on hematoxylin-eosin staining, was identical to that of the sigmoid colon cancer found in 1993 and the “intrahepatic cholangiocarcinoma” found in 2002. Furthermore, the macroscopic types of the three specimens were similar. Immunohistochemically, the cytokeratin and mucin core protein expression patterns were similar. All three specimens were negative for cytokeratin 7 (CK7) and positive for cytokeratin 20 (CK20) (Fig. 7). This expression pattern was indicative of metastatic adenocarcinoma, which most often arises from the colon or rectum [5]. In addition, neighboring biliary epithelium was CK7-positive and CK20-negative. All three specimens were weakly positive for Mucin2 (MUC2) but negative for Mucin1 (MUC1), Mucin5AC (MUC5AC), and Mucin6 (MUC6). In reviewing the case history, we recalled that a very low-density mass had been depicted in the intrapancreatic bile duct in October 2005 and it had gradually enlarged over several years (Fig. 8). We concluded that the intrabiliary tumor found in 2002 was a liver metastasis arising from the sigmoid colon cancer and showing intrabiliary growth and that the intrapancreatic bile duct tumor originated from the spontaneous shedding of the biliary tumor thrombus or from the implantation of tumor cells as a complication of percutaneous transhepatic biliary drainage. Twelve months have passed since the last surgical intervention, and there has been no sign of local recurrence or distant metastasis.

## Conclusions

Morphologically, liver metastases from colorectal carcinoma usually appear as regular or irregular rounded masses. Intrabiliary growth of liver metastasis originating from a colorectal carcinoma is a rare manifestation of metastatic liver carcinoma [1] that resembles intrahepatic cholangiocarcinoma. There have been several reports of this type of liver metastasis, which seems to be less aggressive than usual liver metastasis of colorectal carcinoma [1, 6–9]. Okano et al. [6] reported that recurrence-free survival was significantly longer in cases of colorectal liver metastasis with macroscopic intrabiliary tumor growth than in cases of colorectal liver metastasis without this feature. Likewise, Kubo et al. [7] reported that colorectal liver metastasis showing macroscopic intrabiliary extension was less aggressive and had a better prognosis than that of colorectal liver metastasis without macroscopic intrabiliary extension. Sugiura et al. [1] recommended anatomic liver resection for colorectal liver metastasis with intrabiliary tumor growth, despite the common performance of nonanatomic liver resection for liver metastasis. An aggressive surgical strategy may result in improved outcomes in such cases. In our patient, a remnant intrapancreatic bile duct metastasis was resected 12 years and 4 months after major hepatectomy with skeletonization of the hepatoduodenal ligament and biliary tract reconstruction was performed.

Intrapancreatic bile duct metastasis from colorectal carcinoma is rare, with only a few cases reported [2, 3]. Sano et al. [2] described an intrapancreatic bile duct metastasis that was thought to have arisen from cancer cell implantation; spontaneous shedding of cancer cells may have occurred. Another possibility was that because the patient underwent preoperative biliary decompression, the percutaneous transhepatic biliary drainage (PTBD) catheter may have grazed the intrabiliary tumor, injured the biliary mucosa, and caused cancer cell implantation. The rate of percutaneous transhepatic biliary drainage catheter tract recurrence has been shown to be significantly high in patients treated for cholangiocarcinoma of the macroscopic papillary type or histologic well-differentiated adenocarcinoma type [10]. A papillary, well-differentiated cholangiocarcinoma is thought to be fragile and quite susceptible to collapse. Although ours was a case of recurrent colonic cancer rather than cholangiocarcinoma, the tumor was a papillary, well-differentiated adenocarcinoma. Thus, we suspected that the intrapancreatic metastasis had arisen from cancer cell implantation (via spontaneous shedding or as a complication of PTBD), not from hematogenous intrapancreatic bile duct metastasis.

In such circumstances, the metastasis is not systemic; it is a local disease. Therefore, in selected patients with recurrent colorectal cancer, complete surgical resection may be the best therapeutic option for obtaining a good outcome. However, the pancreatoduodenectomy we performed after major hepatectomy with skeletonization of the hepatoduodenal ligament and biliary tract reconstruction was extremely difficult because of the marked adhesion caused by the previous operation. Sano et al. [2] stressed the importance of preserving the previous bilio-enteric anastomosis in a similar situation. We were able to preserve the previous bilio-enteric anastomosis, and we performed subtotal stomach-preserving pancreatoduodenectomy with R0 resection. This aggressive surgical approach for the recurrence of intrapancreatic bile duct metastasis from colon cancer may improve survival, but it is technically demanding and should be performed by an experienced hepatobiliary surgical team.

It can be difficult to differentiate between liver metastasis with intrabiliary growth and intrahepatic cholangiocarcinoma, not only on the basis of preoperative imaging findings but also on the basis of histologic examination with hematoxylin-eosin staining. Immunohistochemical staining proved to be very useful for a precise diagnosis in our case. The immunohistochemical staining for CK7 and CK20 was essential to the diagnosis. The effectiveness of immunohistochemical staining in similar situations has been reported [11, 12]. Differential diagnosis of a liver tumor with intrahepatic bile duct dilatation is very important for determining the therapeutic strategy. Thus, careful examination is needed to diagnose the intrahepatic biliary dilatation, especially for patients with a history of carcinoma in the digestive tract. In addition, in recognition of its indolent biologic behavior, long-term postoperative follow-up should be considered in cases of colorectal liver metastasis with intrabiliary tumor growth.

## Consent

Written informed consent was obtained from the patient for publication of this case report and any accompanying images. A copy of the written consent is available for review by the Editor-in-Chief of this journal.

## Abbreviations

CK: cytokeratin
CT: computed tomography
ISGPF: International Study Group on Pancreatic Fistula
MUC: mucin
PTBD: percutaneous transhepatic biliary drainage
